# The relation between the gut microbiome and osteoarthritis: A systematic review of literature

**DOI:** 10.1371/journal.pone.0261353

**Published:** 2021-12-16

**Authors:** Emanuele Chisari, Marjan Wouthuyzen-Bakker, Alex W. Friedrich, Javad Parvizi

**Affiliations:** 1 Rothman Orthopaedic Institute at Thomas Jefferson University, Philadelphia, Pennsylvania, United States of America; 2 Department of Medical Microbiology and Infection Prevention, University of Groningen, University Medical Center Groningen, RB, Groningen, Netherlands; Washington State University - Spokane, UNITED STATES

## Abstract

**Background:**

Along with mechanical and genetic factors, emerging evidence suggests that the presence of low-grade inflammation has a role in the pathogenesis of osteoarthritis (OA) and seems to be related to the microbiome composition of the gut.

**Purpose:**

To provide evidence whether there is clinical or preclinical evidence of gut-joint axis in the pathogenesis and symptoms of OA.

**Methods:**

An extensive review of the current literature was performed using three different databases. Human, as well as animal studies, were included. The risk of bias was identified using ROBINS and SYRCLE tools, while the quality of evidence was assessed using GRADE and CAMADARES criteria.

**Results:**

A total of nineteen articles were included. Multiple animal studies demonstrated that both obesity, and high-fat and high-sugar diets resulted in a gut dysbiosis status characterized by increased Firmicutes/Bacteroidetes (F/B) phyla ratio and increased permeability. These changes were associated with increased lipopolysaccharide serum levels, which consequently resulted in synovitis and OA severity. The administration of pre-and probiotics partially reversed this bacterial composition. In addition, in human studies, a decreased amount of gut Bacteroidetes, subsequent increased F/B ratio, have also been observed in OA patients.

**Conclusions:**

Our review confirms preliminary yet sound evidence supporting a gut-joint axis in OA in primarily preclinical models, by showing an association between diet, gut dysbiosis and OA radiological severity and self-reported symptoms. Clinical studies are needed to confirm these findings, and to investigate whether interventions targeting the composition of the microbiome will have a beneficial clinical effect.

## Introduction

Osteoarthritis (OA) is the most common degenerative joint disease [1]. Though it may develop in any joint, it predominantly affects diarthrodial joints (mainly knees, hands, or hips) and, following disease progression, it ultimately leads to joint failure [2]. The understanding of the pathophysiology is still evolving. Among the causes, mechanical factors and genetic factors have been classically shown to play a significant role in the development of OA [3, 4]. Despite the fact that OA traditionally has been considered as “non-inflammatory,” low-grade inflammation seems to play an important role in the initiation and propagation of OA. Emerging evidence suggests that this inflammatory state is triggered by the gastrointestinal microbiome [5].

The gastrointestinal microbiome is defined as the sum of all the genetic material of all microbiota that is present in the gut, their metabolic byproducts, and comprises more than 3 million genera and about 5 thousand bacterial species [6, 7]. While much more has to be explored to better define this biological niche, what is known is that each individual is thought to feature a unique microbiome composition [8] that acts as a fingerprint of each individual subject. Taxonomic studies report that Firmicutes (including *Lactobacillus*, *Bacillus*, *Clostridium*, *Enterococcus*, and *Ruminicoccus* genera) and Bacteroidetes are the main phyla, representative of 90% of the total gut microbiome in people with an unspecific diet [9]. Many studies suggest that several intestinal [10] and extra-intestinal diseases [11] are associated with specific bacterial motifs and a modification and disbalance of the microbiome composition (the concept of dysbiosis). Although a causal relationship between dysbiosis and pathophysiology is still under discussion, a direct correlation seems to be plausible, as demonstrated in several chronic conditions and specific pathobionts in a mouse model [12]. Moreover, diseases that have been traditionally considered autoimmune-based are considered to be the results of a significant interaction between innate and adaptive immune response the overall sum of microorganisms, including fungi, virus and their metabolites, in the gut [12].

The joint environment is traditionally considered sterile. However, preliminary findings suggest that the gut-joint axis exist and is particularly active during the neo-angiogenesis phase of OA [13] when bacteria and bacterial products have a preferential gate from the blood to the cartilage and subchondral bone that usually is less vascularized [14–16]. These bacterial elements xmay affect the epigenetic landscape of chondrocytes [17] and prime the innate immune response in the joint via Toll-like receptors signalling [5]. In addition, a recent study [18] suggests that a microbiome may exist inside the OA knee and hip joint as well. The overall ecological interaction among these microbial patterns is still unknown.

Previous studies and methods have underestimated the impact of the microbiome and its products in the pathogenesis of OA [19]. We, therefore, asked whether there is clinical or preclinical evidence of a gut-joint axis in the pathogenesis of OA.

## Methods

### Search strategy

The systematic review was conducted according to the guidelines of the Preferred Reporting Items for Systematic Reviews and Meta-Analyses (PRISMA) [20]. A comprehensive search was performed using three electronic medical databases (PubMed, EMBASE, and Cochrane Library) by two independent authors (GA and VI) from January 2020 to April 2020. To achieve maximum sensitivity we combined the search terms “gut microbio* OR gut OR streptococcus OR microbiome” with some terms related to osteoarthritis and inflammation such as “arthritis OR osteoarthritis OR inflammation OR synovial OR synovitis” and typical anatomical landmarks of disease “hip OR knee” as either keywords or Medical Subject Heading (MeSH) terms. The search strategy was repeated during the peer-review process to further check for newly published articles (October 14th, 2021). The reference lists of all included articles, previous reviews on the topic, and top hits from Google Scholar were reviewed to identify further potentially relevant studies, which were assessed using the inclusion and exclusion criteria. To avoid overlap with other ongoing review studies, we searched PROSPERO for any similar reviews.

### Selection criteria

The gut microbiome is affected by several factors, including dietary patterns [21], pro- and prebiotics, and drugs (antibiotics, proton-pump inhibitors, non-steroidal anti-inflammatory drugs [22, 23]). Inclusion criteria were (1) both clinical and preclinical studies with any level of evidence (2) published in peer-reviewed journals in English, (3) investigated the role of gut microbiome, with or without dietary interventions, and OA pathogenesis or related-symptoms. We excluded studies in which data was not accessible or missing, or those without an available full-text article. We also excluded duplicates and studies with poor scientific methodology as per risk of bias assessment. Abstracts, case reports, conference presentations, reviews, editorials, and expert opinions were excluded.

Two authors (EC and MW) performed the search and evaluated the articles independently. An experienced researcher in systematic reviews (JP) resolved cases of doubt. 465 articles were retrieved from the three databases after removal of duplicates. First, each of the two investigators read the abstracts of all articles, selected relevant articles according to the inclusion and exclusion criteria, and defined a list of articles for full text reading. Then, the two investigators proceed to full text reading, selected the articles eligible for inclusion and compared the results. An experienced researcher in systematic reviews (JP) resolved cases of doubt. After four weeks, the same studies were reread to ensure the investigators agreed about article selection. There was no disagreement among the investigators. One investigator (EC) extracted data from the full-text articles into an Excel spreadsheet with structured tables to analyze each study descriptively. Doubts and inconsistencies were grouped and resolved. Ultimately, 19 articles were found eligible to be included in the study.

### Data extraction and criteria appraisal

**Fig 1:**
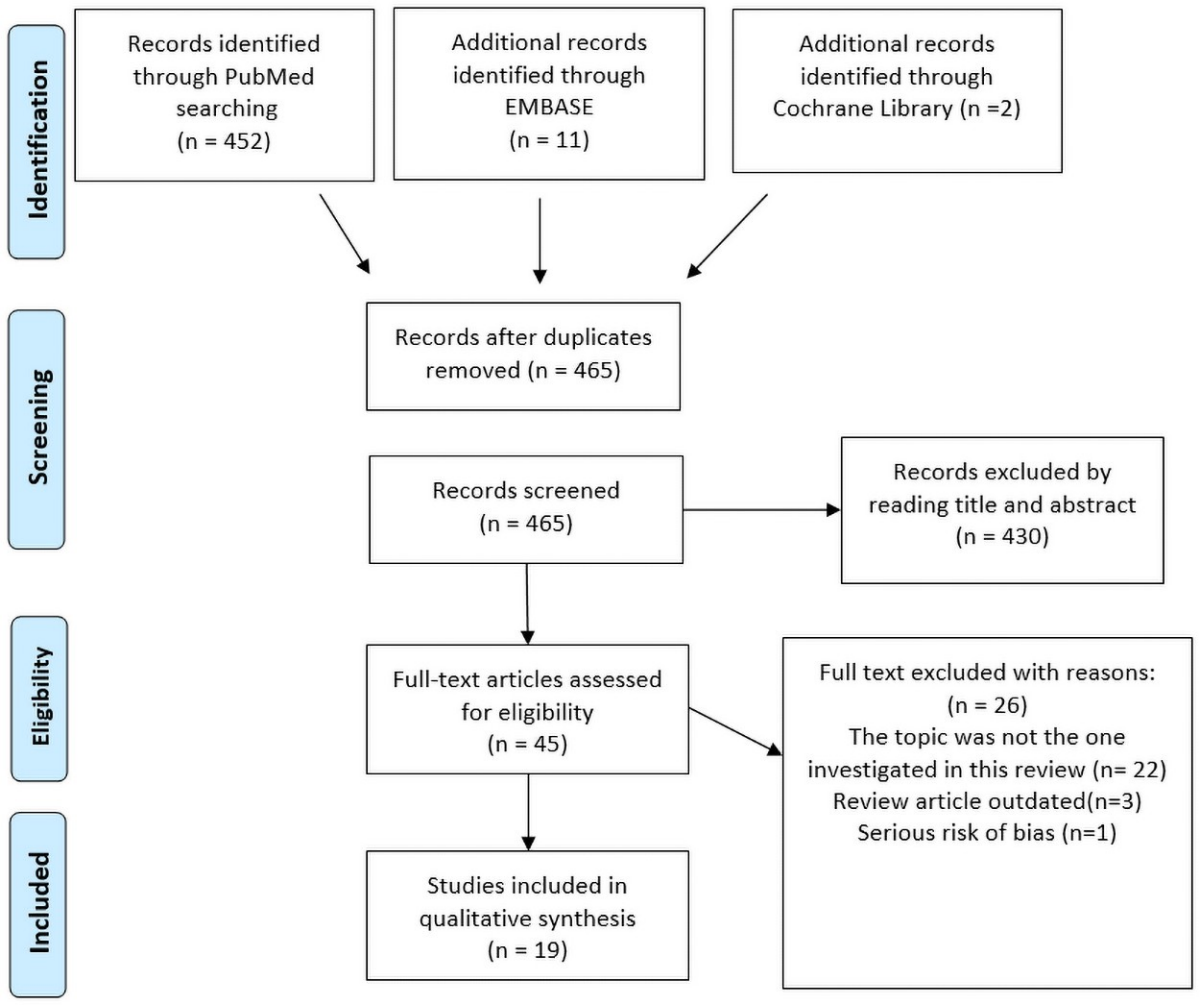
A PRISMA flowchart showing the selection process.

Data were extracted from article texts, tables, and figures using the Population, Intervention, Comparison, Outcome framework [24] (PICO) and included the title, year of publication, study design, sample size, study population, patient characteristics, intervention and comparator group (if applicable), outcomes, findings, and conclusions. Two investigators (GA and GG) independently reviewed each article. Discrepancies between the reviewers were resolved by discussion and consensus (Fig 1). After extraction, the data was considered of heterogenous nature both by study design, measure, and method of assessment. Therefore, a descriptive analysis approach was preferred to a metanalysis.

### Risk of bias assessment

Risk of bias assessment of non-randomized clinical studies was performed according to the ROBINS risk of bias tool [25] for non-randomized study and RoB 2.0 [26] for randomized studies. This assessment used “low,” “moderate,” and “high” as judgment keys. “Low” indicated a low risk of bias, “moderate” indicated a moderate risk of bias, and “high” indicated a high risk of bias. The assessment was performed by two authors (EC and AWF) independently. The inter-rater agreement was 90%. Any discrepancy was solved by consensus. The risk of bias assessment for preclinical studies was instead performed using the SYRCLE’s tool [27]. A score ranging from 0 (lowest) to 10 (highest) was used to classify potential study bias. Two investigators evaluated the studies independently (EC and AWF) with a 95% inter-rater agreement. 12 studies were retrieved after full-text reading and were assessed for the risk of bias. One clinical study was excluded because of a serious risk of bias. Three clinical studies were assessed to have a moderate risk of bias, and one article was excluded because of a serious risk of bias. One randomized clinical study was assessed to have “some concerns” according to the RoB tool. The preclinical animal studies were assessed as having a low to moderate risk of bias (mean SYRCLE score 3.9, range 3–6). (Supplementary Material) (AUTHOR NOTE: weblearn does not allow for supplementary material submission, happy to share by email).

### Study quality assessment

The quality of clinical studies was assessed using the GRADE method [28]. Each study was classified as “low”, “moderate” or “high” according to the quality of evidence. With the exception of one study that featured a low quality of evidence (already excluded after the risk of bias assessment), all the studies ranked “high”.

The Collaborative Approach to Meta-Analysis and Review of Animal Data from Experimental Studies (CAMADARES) checklist [29, 30] was used to assess the quality of the preclinical studies (n = 10) (Supplementary Material) (AUTHOR NOTE:weblearn does not allow for supplementary material submission, happy to share by email). The assessment was performed independently by two authors (EC, AWF) with an inter-rater agreement of 94%. Each study was assessed and scored on a scale from 0 (lowest) to 10 (highest) points. The overall quality was moderate among the included studies (mean CAMADARES score 4.45, range 3–6).

## Results

### Study characteristics

19 studies were included in the final analysis, of which 10 were on animal models [31–38], 8 were human clinical or ex vivo studies [5, 18, 19, 38–47], and one was based on analysis of publicly available databases [40].

Concerning the human clinical trials, 3 were non-randomized observational studies [5, 18, 19], and 3 were randomized clinical trials [41–43]. The main features of the studies included are reported in Table 1. Data was retrieved from 1,548 individuals, of which 58.2% were women. The population was aged between 50 and 65 years. The models investigated in the preclinical studies were adult mice [33, 36, 48–50], and adult rats [31, 34, 35] (Table 1).

**Table 1:** The main findings of the included studies.

AUTHOR (DATE) [REFERENCE]	SUBJECTS NUMBER	SEX	Assessment of OA changes	MEAN AGE (STANDARD DEVIATION)	STUDY DESIGN	MAIN FINDINGS
Boer CG. et al. (2019) [5]	(n = 1427) humans	57.5% female (n = 821).	WOMAC aKellgren-Lawrence radiographic OA severity scores	56.9 (5.9)	population-based cohort study	A robust association between a greater abundance of *Streptococcus* spp. in the gastrointestinal microbiome and WOMAC-pain scores.
Collins KH. et al. (2015) [35]	(n = 32) Sprague Dawley rats	male	Modified Mankin Criteria at sacrifice	8–12 weeks		Increased OA in DIO animals is associated with greater body fat, not body mass. The link between gut microbiota and adiposity-derived inflammation and metabolic OA warrants further investigation
Dunn CM. et al. (2020) [18]	n = 75 humans (n = 34 Hip OA, n = 21 knee OA, n = 20 controls) n = 23 mice (n = 15 C57BL/6, n = 8 MRL/Mpj)	38 women (18 Hip OA, 12 Knee OA and 8 controls) 23 male mice	OARSI score	Hip OA 65 (2) Knee 59 (2) Mice 11 weeks	observational study	Evidence of microbial nucleic acid signatures in human and mouse tissue: increase in gram negative constituents in OA compared to controls and demonstration that knee and hip are distinct both with or without OA
Huang ZY. et al. (2016) [19]	(n = 25) humans	18 women	Kellgren/Lawrence grade, OARSI score and WOMAC score	62.4 (15.8)	population based cohort study	role of LPS in the pathogenesis and severity of structural abnormalities and symptoms of knee OA is strongly supported
Rios JL et al. (2019) [31]	(n = 56) Sprague Dawley rats	male	Modified Mankin score and OARSI	12-weeks		Prebiotic fiber supplementation, aerobic exercise, and the combination of the two interventions completely prevented knee joint damage that is otherwise observed in this rat model of obesity.
Schott EM et al. (2018) [32]	not specified	not specified	OARSI score and histomorphometric analysis using the OsteoMetrics system	19 weeks		the OA of obesity is an inflammatory process driven by obesity-related dysbiosis of the gut microbiome that can be treated by restoring a healthy microbial community using the indigestible prebiotic fiber oligofructose
Panicker S. et al. (2009) [33]	(n = 20) C57BL/6 mice	female	Histological analysis in a papain injected mices	10-week		Oral GlcN alters the physiology of the liver and mesenteric lymph nodes, which in turn, could indirectly alter the biology of the injured joint
So JS. et al. (2011) [34]	(n = 51) Wistar rats	female	The hind paw withdrawal threshold (PWT) and histological analysis	6–8 weeks		evidence that L. casei could act as a potent nutraceutical modulator for OA treatment by reducing pain, inflammatory responses, and articular cartilage degradation
Coulson S. et al. (2012) [41]	(n = 38) humans	29 female	WOMAC score and the SF-12	58.6 (8.9)	non blinded-randomized clinical trial	nutritional supplements such as GLM and GS may regulate some of the metabolic and immunological activities of the GIT microbiota
Ulici V. et al. (2018) [36]	(n = 55) C57BL/6J mice (36 younger, 13 older, 6 dead)	male	ACS score and the safranin-O staining score	younger 13.5 weeks, older 43 weeks		Results suggest factors related to the gut microbiota promote the development of OA after joint injury.
Guan et al.(2020) [37]	(n = 54) C57BL/6N mices	27 males	OARSI score, biomarkes measuraments, DXA, and CT-scan	All 8 weeks old	self-control experiment for the induction of OA, then multiple groups (6) based on sex and antibiotic administration or not	antibiotic-induced gut microbiota dysbiosis in OA male mice significantly decreased the relative abundance of Bacteroidetes, but for female mice, the relative abundance of Firmicutes was increased significantly compared with that in Con-OA female mic
		27 females				
Hu X.H. (2021) [40]	Data coming from the following publications pmid:30664745 pmid:33462485	NA	DNA and RNA sequencing data	NA	Large cohort study based on previously published data	The study was able to identify several microbial taxa associated with joint OA in humans. This data can be used as reference and guide for future studies
Loeser R.F (2021) [39]	Cases: 50 OA with obesity patients (KL>2) Control: 42 no OA with obesity patients	Cases: 43 Females and 7 males Controls: 26 Females and 16 males	16s sequencing data, plasma LPS and LBP	Cases: 73.7 ± 6.9 Control: 70.8 ± 6.4	Case-control	The lack of differences in the gut microbiota yet increased serum LPS levels suggest the possibility that increased intestinal permeability allowing for greater absorption of LPS may contribute to development of OA associated with obesity
Collins KH et al (2021) [38]	Chow WT n = 22, Chow LD n = 15, HFD WT n = 17, HFD LD n = 8, MEF-R n = 15, WF-R n = 12	NA	16s sequencing data, plasma LPS	Start study at 6–8 weeks, surgery for destabilization of medial meniscus at 16 weeks, assessment at 28 weeks	Case-control	The lack of effect from transplant fat, suggest the presence of causal relationships the gut microbiome and cartilage health, independent of diet or adiposity
Huang ZY et al (2018) [47]	431 knee OA patients from the doxycycline (DOXY) trial	All female	Plasma lipopolysaccharide and lipopolysaccharide	45–64 years	Secondary analysis of a clinical trial	Plasma LBP and sTLR4 were associated with knee OA progression over 16–18 months.
Huang ZY et al 2020 [44]	human healthy controls (OA-METS-, n = 4), knee OA without metabolic syndrome (OA+METS-, n = 7) and knee OA with metabolic syndrome (OA+METS+, n = 9)	all female	Medical history, fecal 16s sequencing and blood samples ELISA assay for inflammatory biomarkers (G-CSF, IL-1β, IL-6, IL-10, IL-17, IP-10, MCP-1 and MIP-1α) and the LPS detection	NA	Ex vivo study	Alterations of Fusobacterium, Faecalibaterium and Ruminococcaceae suggest a role of these particular microbes in exacerbating OA.
Chen J (2021) [45]	57 patients with OA and Sex-matched healthy control	All female	Shotgun metagenomics	65.0 ± 7.7 years	Matched cohort study	Significant alterations in the gut microbial composition and function were observed between the older patients with OA and their controls
Wang Z (2021) [46]	182 stool samples from overweight OA patients (n = 86) and overweight normal people (n = 96)	Cases: 25 males and 61 females Control: Matched cohort	16s sequencing of multiple stool samples	The mean age for the overweight OA patients (between 50 and 72 years of age, n = 25 males, n = 61 females) was 62 years. The mean age for the BMI matched healthy controls (between 50 and 76 years of age, n = 40 males, n = 56 females) was 64 years	Ex vivo matched case-control study	Analysis of the gut microbiome could serve as a non-invasive tool for overweight individuals to evaluate their risk for OA
Jhun JY (2021) [42]	Wistar rats (n = 6)	Male:4	RT-PCR, immunohistochemistry of ex-vivo cartilage	6-week-old	Ex vivo study	L. rhamnosus treatment led to decreased pain severity and cartilage destruction in a rat model of OA
		Female:2				

OA: osteoarthritits; OARSI: Osteoarthritis Research Society International; HF: high fat; LPS: lipopolysaccaride; ACS: cartilage society score; WT:wild tipe; LD:lypodistrophic; MEF-R: LD mice who received a mouse embryonic fibroblast transplant; WF-R: mice who received wildtype fat transplant containing visceral and subcutaneous fat.

Most studies analyzed the relationship between OA and the variations in microbial populations of the gastrointestinal tract or in the local articular cartilage (specifically knee and hip samples) by 16S ribosomal RNA gene sequencing, internal transcribed spacer (ITS) amplification and/or shotgun metagenomics [18]. Overall, all studies showed different level of evidence of the role of the gut-joint axis in the pathogenesis and progression of OA.

One study analyzed, in a model of sex steroid deficiency, the role of specific microbial species and strains (administrated by oral gavage of probiotic supplementation) in the maintenance of intestinal barrier integrity. Metabolic alterations of the microbiome and their correlation to gut permeability were investigated by measuring the serum concentration of lipopolysaccharides (LPS), a component of the outer membrane of Gram-negative bacteria [18, 19, 31, 32, 35, 36]. Diet and obesity were analyzed concomitantly [31, 32, 35] in relation to gut microbiome in mice.

Some studies analyzed the role of other dietary aspects in relation to OA severity: physical exercise [31, 49], the administration of nutritional supplementation [34] and/or prebiotics in rodents [31–34] and humans [41] (Table 1).

### Effects of diet regimens and the gut-joint axis

High-fat diet (HFD) and steroid-deficiency induced obesity are common models of gut epithelium disruption, gut dysbiosis, and low-grade systemic inflammation [31, 35].

When mice were on an HFD, OA-like and inflammatory changes were observed during histological analysis. However, when pre-and probiotics were administered contextually to the food, the inflammatory effects on the gut-joint axis were partially reversed and prevented [31, 35]. Another study, reported similar results with the administration of oligofructose in obese mice with increased gut permeability [32]. However, Collins et al. [38] reported how these changes can happen even independently of HF diet, but based on direct relationship between the microbiome and the OA phenotype. A study tried to replicate what was previously found by investigating the gut microbiome and LPS level in a case-control study of OA vs non-OA human patients [39]. While alpha e beta diversity did not differ among the two groups, the LPS level were strongly associated with OA severity (104.9 ± 45.8 EU/mL vs 61.3 ± 33.9 EU/mL; p<0.0001) highlighting the role of gut permeability in OA.

Few studies investigated the use of glucosamine sulfate in similar models, and while a non-blinded randomized clinical trial [41] found that both Glucosamine sulfate and Green-lipped mussel used as prebiotic, were effective in improving joint function and pain in knee OA patients. However, a preclinical trial showed how glucosamine was effective only in co-administration with *Lactobacillus* supplementation [34], which was shown to prevent the changes even when administered alone.

### Microbial composition changes associated with osteoarthritis

When obesity was induced by administering a HFD in mice models, the most common finding was an increased Firmicutes/Bacteroidetes (F/B) phyla ratio in the gut compared to chow-fed mice [31, 32, 35] (Table 1) (F/B = 5–10 [35]. This finding was found to be strongly correlated with OA severity according to the modified Mankin Criteria, using a histologic/histochemical grading system.

Quantitatively, the F/B ratio increase has shown to be mostly dependent of a decrease in Bacteroidetes phylum population (especially *Bacteroides* and *Prevotella* genera), whereas the total Firmicutes abundance mostly remained the same. However, the Firmicutes composition was different in OA patients, mainly showing a decrease in *Lactobacillus spp*. and an increase in the *Clostridiales* order [35]. The largest OA human population investigating the gut microbiome, showed that the relative most abundant species among *Lactobacillales*, was *Streptococcus spp*., which have shown to represent 20% of *Firmicutes* in OA patients and this was positively correlated with higher WOMAC OA pain scores and lower functionality [5]. These results were validated by replication in an independent cohort and by meta-analysis of the final results. Finally, they presented evidence that this association was driven by local inflammation (synovitis and joint effusion) in the joint assessed through a subgroup MRI analysis in 373 patients. Interestingly, a recent study looking a publicly available sequencing data, used mendelian randomization approach to identify microbial taxa associated to OA in humans [40]. Their finding were in agreement with what previously reported.

## Discussion

**Fig 2:**
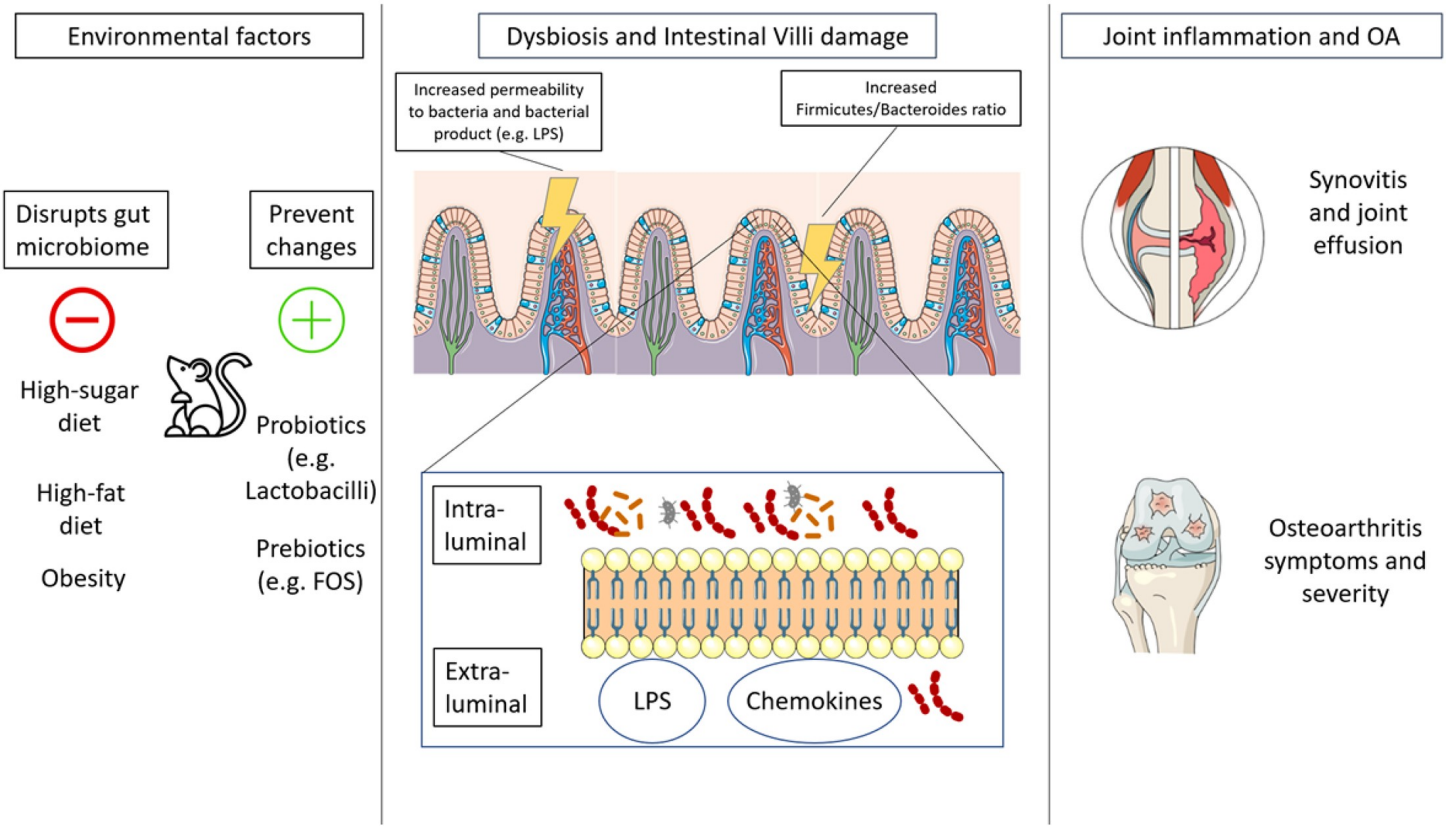
The model of gut-joint axis. FOS: fructooligosaccharides; LPS: lipopolysaccharide; OA: osteoarthritis. In the picture are described the three main actors of the gut-joint axis in OA development. Environmental factors act as either stressor or protectants of gut integrity and microbiota health. If this balance is disrupted, bacterial metabolites and byproducts such as LPS and chemokines pass the gut barrier into the blood flow causing distant damage to diarthrodial joints such as the knee. In the long-term, similar damage together with immune priming can initiative OA changes and affect pain and functionality.

The results of our systematic review demonstrate that a gut-joint axis exist in preclinical and clinical models. We included 15 studies of level 3 and 4 evidence, and one randomized study. Since these designs cannot prove causality, future large longitudinal controlled studies are encouraged to strengthen this concept. To our knowledge, this is the first review exploring the gut-joint axis in an evidence-based systematic fashion (Fig 2).

### Gut permeability as a foundational element of the gut-joint axis

Gut microbiome changes such as bacterial abundance, activity or diversity, are observed in animal models of obesity, and can be either acute or chronic. Indeed, obesity is associated with specific microbiome patterns that ultimately lead to impairment of the gut mucosa and microbiome translocation [51]. All of these changes in the gut are commonly described as gut dysbiosis. Interestingly, together with physical exercise, weight loss is strongly associated with OA control [52]. Both interventions are associated with reduced pain and disease progression [53, 54]. In addition, along with functional food use [33, 41], these interventions have been shown to be effective in reverting the acute microbiome compositional changes that are associated with OA, as depicted in this systematic review.

When gut dysbiosis occurs as a result of an underlying chronic disease (e.g., inflammatory bowel disease), chronic antibiotic treatment, or lifestyle modification (e.g., obesity and metabolic disease), gut permeability is significantly affected [55–60]. It has been demonstrated that a HFD depletes eosinophils in the gut and is associated with an increase in gut permeability [61], reduced tight-junctions molecules, mucus, and antimicrobial peptides production [62]. Moreover, microorganisms are spatially redistributed in the intestine, mainly occupying intervillous/cryptal spaces [62]. If such changes progressively occur, innate immune receptors in the gut become activated by microbial products (such as LPS) and stimulate the production of pro-inflammatory mediators (IL-1beta; IL-17, IL-18) both locally and systemically once similar byproducts reach the systemic circulation [39]. Thus, both inflammatory mediators and bacterial toxins (e.g., LPS) are subsequently translocated into the circulation [57] with priming action on the immune system that has been associated with multiple chronic conditions [63–67] including OA [39].

Together with molecular products, bacterial translocation is possible and raises concerns, particularly in patients with prosthetic implants or artificial valves [68, 69]. Although emerging evidence suggests that bacterial translocation is associated with OA severity in the native joint, it is still unclear whether an increased risk for infection of the joints does exist in obese patients or in patients with inflammatory bowel disease. Ultimately, low-grade inflammation of the gut may lead to transient bacteremia, in which either direct colonization of bacteria to the joint may occur or in which leucocytes and macrophage [32, 70] may act as “Trojan Horse” [68, 69, 71] for bacteria to reach the joint. This concept needs to be further explored in subsequent studies.

### Adaptive and innate immune response

The aforementioned short-term alterations of the gut microbiome composition are associated with worse patient-reported outcome measures and with a more severe radiologic OA classification [5, 19]. Our findings indicate that this is probably due the observed increase in the Firmicutes/Bacteroidetes (F/B) phyla ratio of the gut microbiome [31, 32, 35].

Bacteroidetes produce high levels of *short-chain fatty acids*, which regulate the differentiation of *Treg* cells that are the main actors of the suppression of inflammation in chronic and acute condition [72]. Thus, we propose that the observed clinical findings are due to a decrease in Bacteroidetes, as displayed in OA patients, through the impairment of adaptive immunity and the delicate balance of pro-and anti-inflammatory mechanisms. Also, the abundance of specific strains, such as *Streptococcus spp*. and other gram-negative strains seems to be critical to prime both local and systemic inflammation through LPS- or other metabolites-induced macrophage activation [5] via common inflammatory pathways such as *NFkB* or *MAPK* [73], or by forming active complexes with Lipopolysaccharide-binding-Protein (LBP), laminin-binding protein, and CD14 [19]. Mechanistic evidence was reported with increased CD14 levels (as a surrogate marker for macrophage activation) in the synovium of OA patients compared to healthy controls [74]. Thus, an increase in F/B ratio and *Streptococcus spp*. prevalence are probably able to dysregulate the delicate balance of pro-inflammatory markers [34]. However, two of the studies we included in our analysis [36, 37], reported that gut microbiome dysbiosis alleviates the progression of osteoarthritis in mice. Additionally, an antibiotic-induced gut microbiota dysbiosis in OA male mice significantly decreased the relative abundance of Bacteroidetes, but for female mice, the relative abundance of Firmicutes was increased significantly compared with that in Con-OA female mice [37]. While this further highlights the pivotal role of the microbiota as whole in the development, progression and symptomatology of OA, also induces contradictory conclusion about bacterial diversity and F/B ratio. While many conclusion can be made, we hypothesize that any change that can lead to impaired gut barrier integrity and the release of byproducts such as LPS into the bloodstream can perhaps induce immune priming and adjuvate the initiation and progression of OA disease.

We also hypothesize that bacterial molecules, that can potentially act as biomarkers, are involved in OA inflammation. We encourage further studies on microbial exotoxins, cationic bacterial peptides, and other metabolites, to assess their role in the pathogenesis of OA, their influence on quorum sensing and inter-and intra-species interactions in biofilm-based musculoskeletal infections such as periprosthetic joint infections [75].

### Pain symptomatology and chemokines

Studies show that the gut microbiome is associated with joint pain [76]. The main actors are cartilage degradation products and other damage-associated molecular patterns either through direct neuronal activation of dorsal root ganglia or by indirect neuro-immune signaling acting on immune cells receptors that in turn stimulate neurons amplifying the mechanism. Apart from cytokines and LPS that may partially explain the onset of pain, *chemokines* are also reported as potential critical players [70]. Indeed, chemokines were consistently increased in murine animal models [32, 49].

Current treatments for OA focuses on pain relief, but do not target the pathogenesis of the disease [52]. Since the gut microbiome is modifiable by several factors (dietary intervention, fecal transplant, and future microbiome-targeted therapeutics), the gut microbiome is a promising target for future treatment strategies.

### Limitations

Our findings have several limitations. First of all, despite the consistent data on the association of OA with specific bacterial strains and phyla ratio in the gut microbiome, we caution the reader about the absence of large human studies other than the few reported. To intervene in the gut-joint axis as part of treatment requires a more profound mechanistic understanding of microbiome-host interactions and detailed characterization of the complex community interactions involved. Current studies, despite the overall moderate to high quality of evidence, still do not allow for such conclusions.

Most studies were conducted on animal models, not fully mimicking the complexity of the human microbiome. In addition, the design of all the studies included do not allow for causality conclusions. However, all pieces of evidence analyzed have shown concordance of findings and clinical studies show similar results [5]. Another major limitation of the analyzed studies is the use of different methods to induce obesity in animal models and the heterogeneity of the studied animal models itself. However, all the different animals and the different models used (high fat diet, high sugar diet, sex steroid deficiency) show the same results. Finally, most animal studies only described the alterations of the gut microbiome after specific diet regimens, and by design are not able to describe a clinically relevant microbiome description. Also, despite the studies reported a control group with different diet regiment (e.g. chow-fed), further studies should investigate the alteration of the bacterial strains, and their association with OA, regardless of the diet, maybe using fecal transplantation as validated in other studies [77]. Finally, bacterial genomic plasticity is missing in most of the studies, where under antibiotic—although subinhibitory—pressure, influence of heavy metals from food or drinking water, and nutritional influence, mobile genetic elements drive in-vivo evolutionary events and adaptation processes in the microbiome, its composition and inflammatory activity.

## Conclusion

In conclusion, our systematic review provides evidence for the existence of a gut-joint axis in the pathogenesis OA. The proposed concept starts with disruption and dysbiosis of the normal gut homeostasis, the continuous change of the microbial composition and genomic plasticity for optimal bacterial adaptation to the host environment, followed by both adaptive and innate immune responses due to translocation of bacteria and bacterial products into the circulation toward to the joint. This cascade ultimately leads to low-grade inflammation in the joint and contributes to the pathogenesis of OA. Future studies are needed to further strengthen this hypothesis.

## Supporting information

S1 Checklist PRISMA 2009 checklist. (DOC)

S1 Table CAMARADES CHECKLIST of the included studies. (DOCX)

S2 Table SIRCLE bias assessment of the included studies. (DOCX)

S3 Table ROBINS bias assessment of the included studies. (DOCX)

S4 Table Rob 2.0 bias assessment of the included studies. (DOCX)
