# Supplementary material for: The relation between the gut microbiome and osteoarthritis: A systematic review of literature
Source: PLoS One. 2021 Dec 16;16(12):e0261353. doi: 10.1371/journal.pone.0261353 (PMC8675674; doi:10.1371/journal.pone.0261353)
Supplement: S1 Table — (DOCX) [file pone.0261353.s002.docx]

**S1 Table.** CAMARADES CHECKLIST of the included studies

| **CAMARADES CHECKLIST** | **RiosJL**  **2019** | **Ulici 2018** | **Schott**  **2018** | **Li**  **2016** | **Collins**  **2015** | **Griffin**  **2013** | **Amdekar 2013** | **Mooney**  **2011** | **Panicker**  **2009** | **Joosten**  **2000** | **Joosten**  **2000(2)** | **Guan et al 2020** | **Collins KH 2021** | **Jhun JY 2021** |
| --- | --- | --- | --- | --- | --- | --- | --- | --- | --- | --- | --- | --- | --- | --- |
| **Publication in peer-reviewed journal** | Y | Y | Y | Y | Y | Y | Y | Y | Y | Y | Y | Y | Y | Y |
| **Statement of control of temperature** | N | N | N | Y | N | Y | N | N | N | N | N | Y | N | N |
| **Randomization of treatment or control** | Y | N | N | N | Y | N | N | N | N | N | N | Y | N | N |
| **Allocation concealment** | N | N | N | N | N | N | N | N | N | N | N | Y | N | N |
| **Blinded assessment of outcome** | N | Y | N | N | N | N | N | N | N | N | N | Y | N | Y |
| **Avoidance of anesthetics with marked intrinsic properties** | Y | N | Y | Y | Y | Y | Y | Y | Y | Y | Y | Y | Y | N |
| **Use of animals with hypertension or diabetes** | N | N | N | N | N | N | N | Y | N | N | N | Y | N | N |
| **Sample size calculation** | N | N | N | Y | Y | N | Y | N | N | Y | Y | Y | N | N |
| **Statement of compliance with regulatory requirements** | Y | Y | Y | Y | Y | Y | Y | Y | Y | Y | Y | Y | Y | Y |
| **Statement regarding possible conflict of interest** | Y | Y | N | Y | Y | N | Y | N | Y | N | N | Y | N | Y |
| **Total (on 10)** | **5** | **4** | **3** | **6** | **6** | **4** | **5** | **4** | **4** | **4** | **4** | Y | **3** | **4** |
