# Supplementary material for: The relation between the gut microbiome and osteoarthritis: A systematic review of literature
Source: PLoS One. 2021 Dec 16;16(12):e0261353. doi: 10.1371/journal.pone.0261353 (PMC8675674; doi:10.1371/journal.pone.0261353)
Supplement: S2 Table — (DOCX) [file pone.0261353.s003.docx]

**S2 Table**. SIRCLE bias assessment of the included studies

| **SYRCLE** | **RiosJL**  **2019** | **Ulici** | **Schott**  **2018** | **Li**  **2016** | **Collins**  **2015** | **Griffin**  **2013** | **Amdekar 2013** | **Mooney**  **2011** | **Panicker**  **2009** | **Joosten**  **2000** | **Joosten**  **2000(2)** | **Guan et al. 2020** | **Collins KH 2021** | **Jhun JY 2021** |
| --- | --- | --- | --- | --- | --- | --- | --- | --- | --- | --- | --- | --- | --- | --- |
| **Was the allocation sequence adequately generated and applied?** | Y | N | N | N | Y | N | N | N | N | N | N | Y | N | N |
| **Were the groups similar at baseline or were they adjusted for confounders in the analysis?** | Y | Y | Y | Y | Y | Y | Y | Y | Y | Y | Y | Y | Y | Y |
| **Was the allocation adequately concealed?** | N | N | N | N | N | N | N | N | N | N | N | Y | N | N |
| **Were the animals randomly housed during the experiment?** | N | N | Y | N | Y | Y | N | Y | N | N | N | Y | Y | N |
| **Were the caregivers and/or investigators blinded from knowledge which intervention each animal received during the experiment?** | N | N | N | N | N | N | N | N | N | N | N | Y | N | N |
| **Were animals selected at random for outcome assessment?** | N | N | N | N | N | N | N | N | N | N | N | Y | N | N |
| **Was the outcome assessor blinded?** | N | Y | N | N | N | N | N | N | N | N | N | Y | N | N |
| **Were incomplete outcome data adequately addressed? (*)** | Y | Y | N | N | Y | N | N | N | N | N | N | Y | N | N |
| **Are reports of the study free of selective outcome reporting? (*)** | Y | Y | Y | Y | Y | Y | Y | Y | Y | Y | Y | Y | Y | Y |
| **Was the study apparently free of other problems that could result in high risk of bias? (*)** | Y | Y | Y | Y | Y | Y | Y | Y | Y | Y | Y | Y | Y | Y |
| **Total (on 10)** | **5** | **5** | **4** | **3** | **6** | **4** | **3** | **4** | **3** | **3** | **3** | 2 | **4** | **3** |
