# Supplementary material for: The relation between the gut microbiome and osteoarthritis: A systematic review of literature
Source: PLoS One. 2021 Dec 16;16(12):e0261353. doi: 10.1371/journal.pone.0261353 (PMC8675674; doi:10.1371/journal.pone.0261353)
Supplement: S3 Table — (DOCX) [file pone.0261353.s004.docx]

**S3 Table**. ROBINS bias assessment of the included studies

| **Author et al. (Year)** | **Study design** | **Risk of Bias** | **Inconsistency of results** | **Indirectness of evidence** | **Imprecision** | **Publication bias** | **Large magnitude of effect** | **Dose-response gradient** | **Plausible confounding** | **Quality** | Confounding | Selection of participants into the study | Classification of interventions | Deviations from intended interventions | Missing data | Measurement of the outcome |
| --- | --- | --- | --- | --- | --- | --- | --- | --- | --- | --- | --- | --- | --- | --- | --- | --- |
| Dunn CM et al (2020) | observational study | Low | Not serious | Not serious | Not serious | Not serious | N/A | N/A | No | High | Moderate | Moderate | Low | No information | Low | Low |
| Boer CG et al. (2019) | observational study | Low | Not serious | Not serious | Not serious | Not serious | N/A | N/A | No | High | Low | Low | Low | No information | Low | Low |
| F. Pan et al. (2019) | observational study | High | Not serious | Serious | Undetected | Undetected | N/A | N/A | No | Moderate | Low | Serious | Low | No information | Low | Moderate |
| Huang ZY et al. (2016) | observational study | Low | Not serious | Not serious | Not serious | Not serious | N/A | N/A | No | Low | No information | Moderate | Low | No information | Low | Moderate |
| X. Zhao et al. (2020) | observational study | Low | Not serious | Not serious | Not serious | Not serious | N/A | N/A | No | High | Low | Low | Low | No information | Low | Low |
| P.Yang et al.(2016) | observational study | Low | Not serious | Not serious | Not serious | Not serious | N/A | N/A | No | High | Low | Low | Low | No information | Low | Low |
| G. Ren et al. (2019) | obvservational study | Low | Not serious | Not serious | Not serious | Not serious | N/A | N/A | No | Moderate | Low | Low | Low | No information | Low | Moderate |
| Z. Xu et al. (2019) | observational study | Unclear | Not serious | Not serious | Not serious | Not serious | N/A | N/A | No | Moderate | Moderate | Low | Low | No information | Moderate | Low |
| Loeser RF (2021) | Case-control | Low | Not serious | Not serious | Not serious | Not serious | N/A | N/A | No | High | Low | Low | Low | No information | Low | Low |
| Chen J (2021) | Matched cohort study | Low | Not serious | Not serious | Not serious | Not serious | N/A | N/A | No | High | Low | Low | Low | No information | Low | Low |
|  |  |  |  |  |  |  |  |  |  |  |  |  |  |  |  |  |
