# Supplementary material for: The relation between the gut microbiome and osteoarthritis: A systematic review of literature
Source: PLoS One. 2021 Dec 16;16(12):e0261353. doi: 10.1371/journal.pone.0261353 (PMC8675674; doi:10.1371/journal.pone.0261353)
Supplement: S4 Table — (DOCX) [file pone.0261353.s005.docx]

| **S4 Table**. Rob 2.0 bias assessment of the included studies | | | | | | | | | | | | | | | | |
| --- | --- | --- | --- | --- | --- | --- | --- | --- | --- | --- | --- | --- | --- | --- | --- | --- |
| **Author et al. (Year)** | **Study design** | **Risk of Bias** | **Inconsistency of results** | **Indirectness of evidence** | **Imprecision** | **Publication bias** | **Large magnitude of effect** | **Dose-response gradient** | **Plausible confounding** | **Quality** | Randomization process | Deviations from intended interventions | Missing outcome data | Measurement of the outcome | Selection of the reported result | Overall |
| Coulson S et al. (2013) | non blinded-randomized clinical trial | Low | Not serious | Not serious | Not serious | Not serious | N/A | N/A | No | Moderate | Low | Low | Low | Low | Some concerns | Some concerns |
| Huang ZY (2018) | Secondary analysis of a clinical trial | Low | Not serious | Not serious | Not serious | Not serious | N/A | N/A | No | Moderate | Low | Low | Low | Low | Some concerns | Some concerns |
